# Ecology and epidemiology of rabies in humans, domestic animals and wildlife in Namibia, 2011-2017

**DOI:** 10.1371/journal.pntd.0007355

**Published:** 2019-04-16

**Authors:** Emmanuel H. Hikufe, Conrad M. Freuling, Rauna Athingo, Albertina Shilongo, Emmy-Else Ndevaetela, Maria Helao, Mathews Shiindi, Rainer Hassel, Alec Bishi, Siegfried Khaiseb, Juliet Kabajani, Jolandie van der Westhuizen, Gregorio Torres, Andrea Britton, Moetapele Letshwenyo, Karin Schwabenbauer, Thomas C. Mettenleiter, Nicolai Denzin, Susanne Amler, Franz J. Conraths, Thomas Müller, Adrianatus Maseke

**Affiliations:** 1 Directorate of Veterinary Services (DVS), Ministry of Agriculture Water and Forestry, Windhoek, Namibia; 2 Friedrich-Loeffler-Institut, Institute of Molecular Virology and Cell Biology, Greifswald-Insel Riems, Germany; 3 Chief Veterinarian-North West, Rabies control project Coordinator, Directorate of Veterinary Services (DVS), Ministry of Agriculture Water and Forestry, Ondangwa, Namibia; 4 Epidemiology Division, Health Information and Research Directorate, Ministry of Health and Social Services, Windhoek, Namibia; 5 School of Veterinary Medicine, University of Namibia, Windhoek, Namibia; 6 Central Veterinary Laboratory, Directorate of Veterinary Services (DVS), Ministry of Agriculture Water and Forestry, Windhoek, Namibia; 7 World Organisation for Animal Health (OIE), Science and New Technologies Department, Paris, France; 8 World Organisation for Animal Health (OIE), Sub-Regional Representation for Southern Africa, Gaborone, Botswana; 9 Unterabteilung 32, Federal Ministry of Food and Agriculture, Bonn, Germany; 10 Friedrich-Loeffler-Institut, Institute of Epidemiology, Greifswald-Insel Riems, Germany; University of Glasgow, UNITED KINGDOM

## Abstract

Rabies is a fatal zoonotic disease that causes a heavy burden on societies. Namibia, a country in southern Africa, is aiming at controlling the disease in its main reservoir, the domestic dog. To facilitate the implementation comprehensive information on the ecology and epidemiology of the disease and surveillance is of utmost importance. The study presented assesses the baseline data for both human and animal rabies surveillance in Namibia in recent times and establishes correlations with ecological and socio-economic data in order to provide an up-to-date picture on the epidemiology of rabies in Namibia. For instance, it was important to identify the main drivers in the epidemiology, and whether the control strategy by mass vaccination of dogs is undermined by cycles of rabies in wildlife. Rabies in humans was reported mainly from the Northern Communal Areas (NCAs), with a total of 113 cases from 2011 to 2017, representing an incidence of between 1.0 and 2.4 annual human rabies deaths per 100,000 inhabitants. Kavango, the region with the highest human rabies incidence was also the region with the lowest animal rabies surveillance intensity. Generally, the vast majority (77%) of dog samples originated from communal farm land, followed by urban areas (17%), while only a small fraction (3%) was submitted from freehold farm areas. In contrast, kudu and eland submissions were almost exclusively from freehold farmland (76%) and urban areas (19%), whereas the submission of cattle samples was evenly distributed among freehold farms (46%) and communal farm land (46%). The likelihood of sample submission decreased exponentially with distance to one of the two laboratories. Overall, 67% (N = 1,907) of all samples submitted tested rabies-positive, with the highest positivity rate observed in kudus (89%) and jackals (87%). The transmission cycle of rabies in dogs appears restricted to the northern communal areas of Namibia, whilst rabies in wildlife species is predominately reported from farmland in central Namibia, mostly affecting kudu (*Tragelaphus strepsiceros)* and livestock with a likely reservoir in wildlife canids such as jackals or bat-eared foxes. The analysis confirms the presence of two independent transmission cycles in Namibia with little geographic overlap, thus allowing for a sustainable control of rabies in dogs in the NCAs.

## Author summary

Rabies is a fatal zoonotic disease. It has been recorded in southern Africa including Namibia since the early 19^th^ century. At present, Namibia is aiming at controlling the disease in its main reservoir, the domestic dog. This study assessed the baseline data for rabies surveillance in Namibia from 2011–2017. There are continuous reports of rabies in humans mainly from the Northern Communal Areas, with a total of 113 cases from 2011 to 2017. Among animal rabies submissions, the vast majority of canine samples originated from communal farm land, whereas kudu and eland submissions were almost exclusively from freehold farmland. Cattle samples were submitted evenly among freehold farms and communal land use. Those three species, i.e. dogs, cattle and kudus represent the animal species with the highest numbers of sample submissions and positives. Spatially, the transmission cycle of rabies in dogs appears restricted to the northern communal areas of Namibia, whilst rabies in wildlife species is predominately reported from farmland in central Namibia, affecting mostly the kudu (*Tragelaphus strepsiceros*). Given the spatial overlap of kudu and livestock rabies cases, an independent horizontal transmission cycle among kudus as previously suggested is highly unlikely. Rather, wildlife canids such as jackals or bat-eared foxes could be the reservoir for wildlife rabies in Namibia.

## Introduction

**Fig 1:**
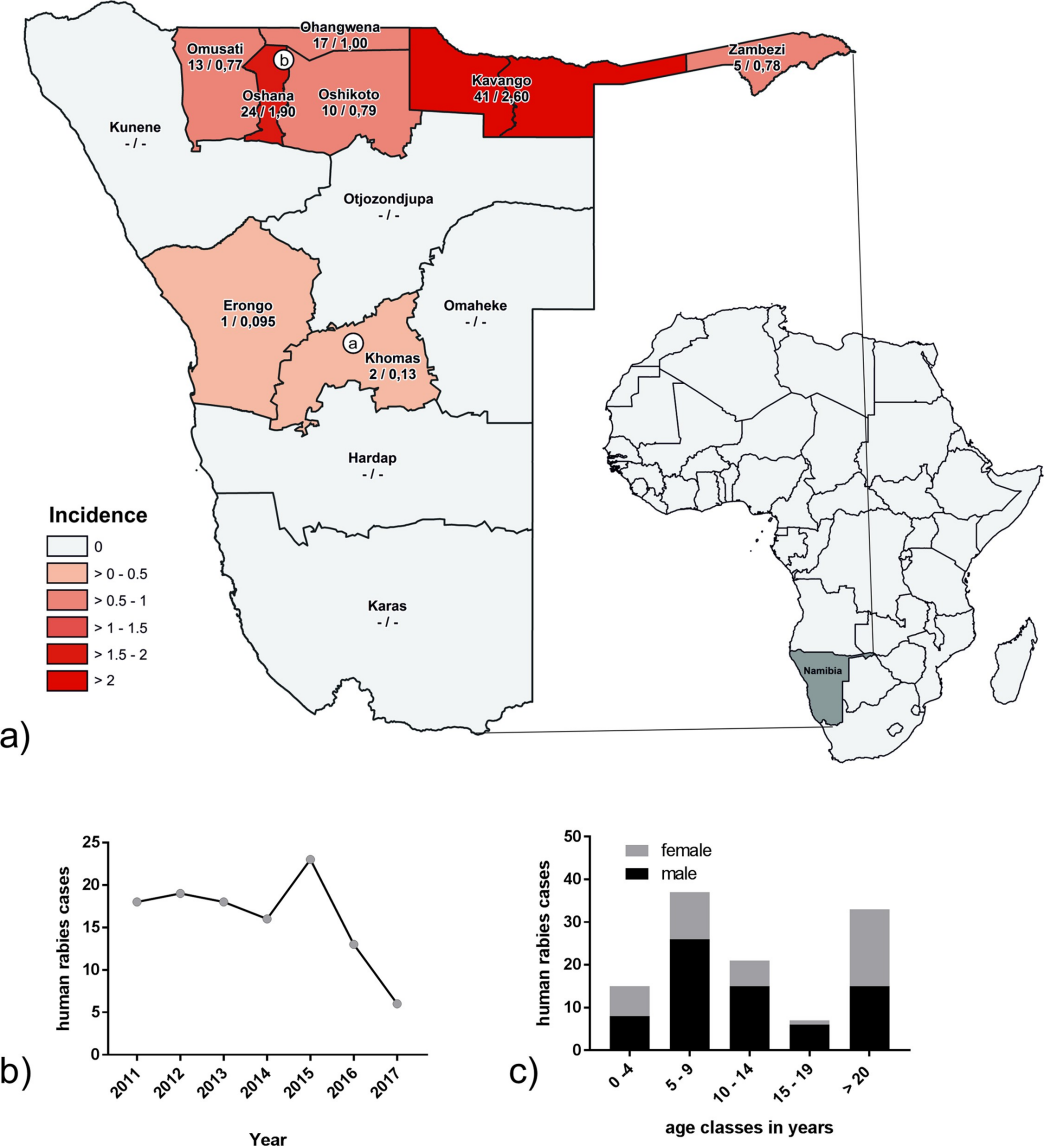
**a) map depicting the location of Namibia in Africa (right) and the boundaries and names of the Namibian districts and the veterinary cordon fence (dark grey).** The color scale indicates the average rabies incidence per 100,000 people and per region. The locations of the laboratories, i.e. the CVL in Windhoek (a) and in Ondangwa (b) is shown. The absolute number of cases per regions and the rabies incidence per 100,000 people is also provided in brackets. Below: temporal trend (b) and the distribution per age class and gender (c) of human rabies cases in Namibia, 2011–2017.

Rabies is a fatal viral infection of mammals. The causative agents are members of the Lyssavirus genus, of which rabies virus (RABV) is the prototype [1, 2]. Domestic dogs cause over 95% of all estimated 59,000 human rabies deaths every year with the highest burden of disease in parts of Asia and Africa [3]. As with the entire continent, rabies is also endemic in Namibia. Situated in the south-western corner of the African continent (Fig 1), Namibia is a large country of about 824,116 square kilometers, and home to about 2.1 million people [4]. With a Human Development Index (HDI) of 0.640 the country is in the medium human development category [5]. Although the contribution of agriculture to the gross domestic product by about 3–4% is low [6], agriculture plays either directly or indirectly an important role. It is mainly based on livestock farming, i.e. cattle, sheep and goats under various management systems. While subsistence farming prevails in the North of Namibia covering about 41 per cent of the total land area and accommodating about 60 per cent of the population, commercial farms predominate in central and southern Namibia. Due to incursions of Food and Mouth Disease, a game- and livestock-proof veterinary cordon fence was built in 1961/1962 that is still functional today [7]. The area north of this fence is referred to as the “Northern Communal Areas” (NCAs). Namibia also supports in excess of two million head of game, which attracts tourists and trophy hunters, is sold as live animals, or is harvested for commercial meat production and for on-farm use [8].

Rabies has first been recognized in Namibia in the beginning of the 19th century. Early reports suggest an introduction by ships from Europe and by a cross-border spread of the disease in the NCAs (reviewed in [9]). Laboratory confirmation indicated that rabies was present both in dogs in the NCAs as well as in other wildlife reservoirs, eventually causing spill-over infection into domestic and wildlife ruminants [10, 11]. Particularly, rabies in the greater kudu (*Tragelaphus strepsiceros*) became a problem. The greater kudu is a member of the Tragelaphine antelopes, a group that is more closely related to cows than to other antelopes. It is a woodland antelope found throughout eastern and southern Africa [12], which, based on genetic criteria, may be further divided into four subspecies with the Zambezi kudu (*T*.*s*. *zambesiensis*) occurring throughout Namibia [13]. However, the delineation according to the phylogenetic species concept (PSC) is controversially discussed [14, 15].

Despite its wide distribution, the greater kudu (elsewhere in the manuscript referred to as kudu) population is sparse in most areas except for Namibia where its density is highest in Africa [12]. First cases of rabid kudus were reported from central Namibia near Windhoek in the early 1970s [16] and the disease subsequently spread northwards to all the major habitats of kudus in the country, including the Etosha National Park. During the first epidemic that lasted from 1977 to 1986, an estimated 50,000 Kudus, approximately 20% of the total population in Namibia, had succumbed to rabies [10, 11]. It was speculated that horizontal transmission among kudus could be the reason for the observed epidemic waves [10, 17–20] and this was partly supported by phylogenetic analyses [21, 22]. Additionally, recent experimental studies support the possibility of onward horizontal transmission among kudu [23].

While rabies in kudus primarily represents a threat to agriculture and tourism since human contacts are exceptional, it is dog-mediated rabies that contributes the most to costly post exposure prophylaxis (PEP) in humans and eventually, if PEP is not administered in time, human fatalities. In Namibia, continuous reports of human rabies cases in the NCAs led the country to develop a sustainable national control strategy towards dog-mediated human rabies elimination [24]. This initiative was supported by international organizations such as the World Organisation for Animal Health (OIE) and the Global Alliance for Rabies Control (GARC) and strives to contribute to the global goal of ending human dog-mediated deaths by 2030 [25]. To facilitate the implementation of the National Rabies Control Strategy comprehensive information on the complexity and incidence of rabies is of utmost importance. The study presented here aims at assessing the baseline data for both human and animal rabies surveillance in Namibia in recent times and at establishing correlations with ecological and socio-economic data in order to provide an up-to-date picture on the ecology and epidemiology of the disease.

## Material & methods

### Data on human rabies

Human rabies surveillance data were retrieved from the epidemiological database of the Ministry of Health. Rabies cases were initially reported from hospitals based on clinical observation.

### Animal rabies surveillance

Rabies is a notifiable disease in Namibia according to the Animal Health Act 1 of 2011. The competent authority in control of animal health, veterinary public health, animal movement and animal disease control is the Directorate of Veterinary Services (DVS) within the Ministry of Agriculture, Water and Forestry (MAWF). Surveillance in animals is based on the reporting of all suspected cases in any species to an official of the DVS, recording of all relevant information on a specific Disease Report Form (DRF) and submission of the latter to the Epidemiology Section of the DVS. Samples of incriminated animals are to be submitted to one of the competent laboratories of Namibia. Dog samples are also submitted when associated with a human bite. Specifically, dog bite cases and potential rabies exposures are to be reported to the nearest state veterinary official who will assess the rabies vaccination status of the animal by way of its vaccination certificate. If the status cannot be verified, the dog is quarantined, whereas if the animal shows clinical signs of rabies, it is euthanized. Samples are then submitted for laboratory confirmation, a letter signed by the diagnostician is sent to the dog bite victim and rabies PEP is provided to the individual free of charge at a clinic in the area. In this study, rabies surveillance data for Namibia were retrieved from the central animal disease database established at the DVS, for the years 2011–2017. Rabies surveillance data is available both as individual and cumulative datasets. The datasets comprised information on the date of submission, farm name, number of sick animals, number of dead animals, number of animals at risk, rabies diagnosis, differential diagnosis, region, and Gauss-Krüger (geographic) coordinates.

### Diagnostic assays

Laboratory confirmed surveillance data originate from the Central Veterinary Laboratory (CVL), Windhoek, and the Regional Laboratory (RL), Ondangwa. For all samples, the standard Fluorescent Antibody Test (FAT) was used following WHO and OIE recommended protocols [2, 26]. Occasionally, the Direct Rapid Immunohistochemical Test (dRIT, [27, 28]) was additionally applied for rabies routine diagnosis at the CVL Windhoek in order to provide validation data for its broader use.

### Data analysis

Prior to analyses, surveillance data concerning rabies in animals of the observation period (2011–2017) were classified as follows: animals, of which samples had been submitted for laboratory confirmation, were classified according to the test result as positive or negative; animals, which had been found dead or were considered suspect without subsequent laboratory confirmation of rabies, were classified as “suspect cases”. Subsequently, the data were analyzed by means of a geographical information system using ArcGis 10.3 (ESRI, Greenland, USA)

As information on the origin of submissions was incomplete for a number of datasets, such data sets had to be corrected manually. To this end, data was georeferenced on the basis of an available GIS layer of farm territories in Namibia. Precise and approximate sampling locations in Namibia were visualized at the highest spatial resolution available, e.g. farm level or veterinary inspection point at crush pens, i.e. a passage in a fence with one narrow end to handle large domestic animals, such as cattle or sheep. To link rabies surveillance with ecological and socio-economic data, highly detailed freely available geodata for Namibia on human population density, livestock (cattle, sheep, goat) and wildlife densities (kudu) were obtained from different official sources (S1 Table). For epidemiological GIS analyses, rabies surveillance data were stratified according to animal species, year, region and laboratory test results with each extracted data record considered as a single animal. Data was visualized using graphPad Prism Version 7.00 for Windows (GraphPad Software, La Jolla, California, USA).

### Statistics

Further statistical analyses were performed using the software R, version 3.4.1 [29]. For descriptive comparisons of the frequencies of positive test results between factors: species, time interval in years, region of Namibia and season period, absolute and relative frequencies as percentages and corresponding 95% confidence intervals (CIs) were summarized. Univariate analysis of potential associations between positivity rates and different factors was carried out using Fisher’s exact test [30]. To evaluate the likelihood to test rabies positive and adjust for potential confounders, multivariate stepwise logistic regression models were applied with model selection by Akaike information criterion (AIC) [31], stratified for each region. Results were reported as Odds Ratios (ORs) with 95% CIs and two-sided Wald-test p values. The local significance level was set to 0.05. Results were considered as hypotheses-generating.

Additionally, a spatial scan statistic ([32]; http://www.satscan.org/) was used to assess spatial clustering. In this analysis, the test-positive samples represented the cases and the test-negative the controls. Therefore the Bernoulli model was chosen when running the scan statistic. As recommended [33], the maximum window size was set to capture up to 50% of the events (cases and controls).

## Results

### Human rabies surveillance

Rabies cases in humans are predominantly reported from the NCAs, with Kavango, Oshana and Ohangwena regions having the highest case numbers and incidences, the latter ranging from 1.0 and 2.4 per 100,000 inhabitants and per year on average. In contrast, human rabies cases in central and southern Namibia appear to occur sporadically (Fig 1A). Rabies cases have been above 16 cases per year from 2011 until 2015 with a maximum of 23 cases observed in 2015 (Fig 1B). The annual numbers of the two recent reporting years are below the previous average of reported cases. Of the total number of 113 cases, the majority (67%) were children and teenagers below 16 years of age, peaking at 5–9 years (Fig 1C). In all age classes, males were overrepresented (average 62%), with highest proportion of males found among the group aged 15–19 (Fig 1C).

### Animal rabies surveillance

**Fig 2:**
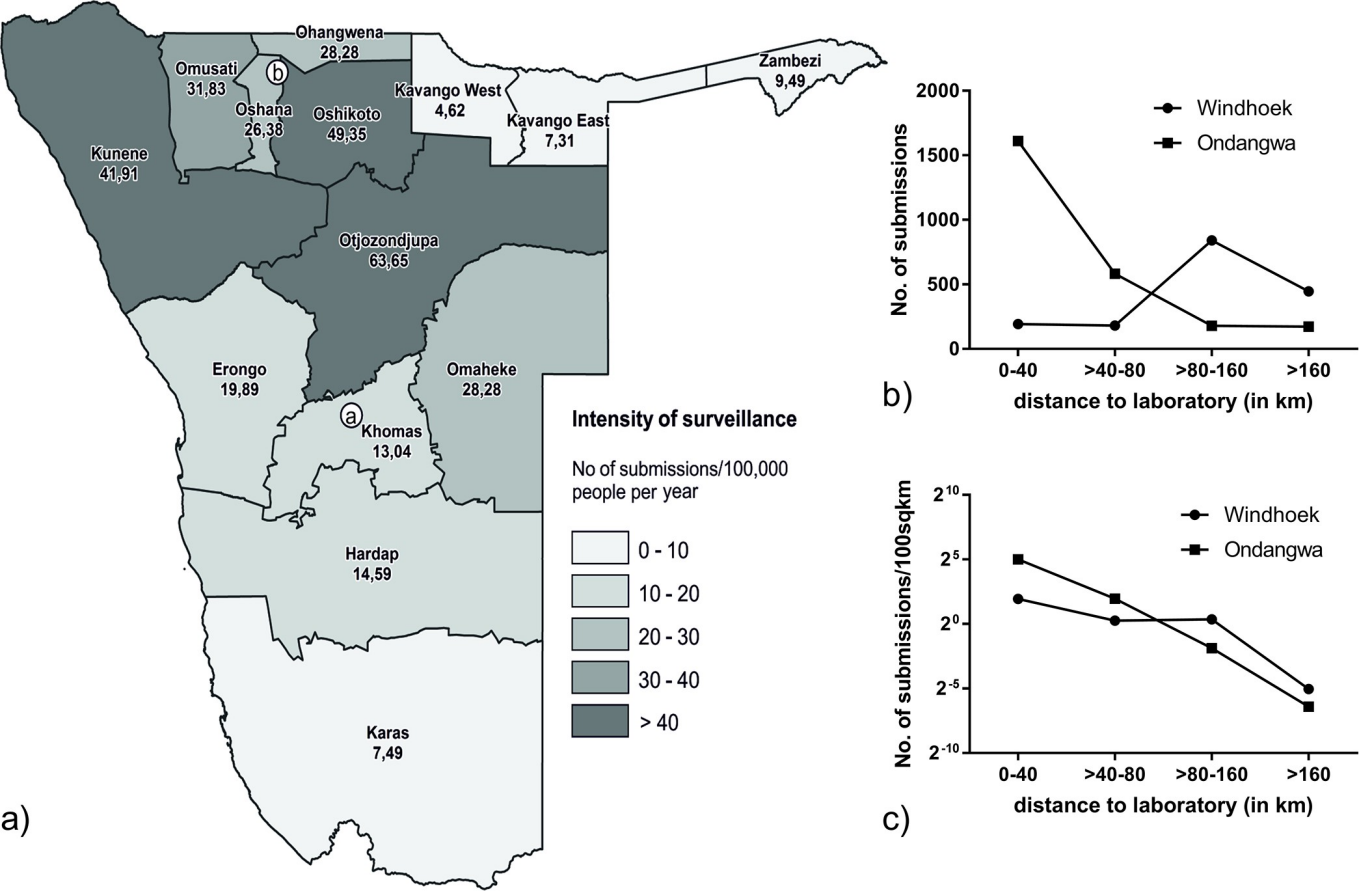
**Surveillance intensity (number of animals submitted for testing per 100,000 inhabitants per year) per region (a).** To the right: Absolute numbers of submissions to the laboratories in Windhoek and Ondangwa per distance (b). Because the area as a potential origin of submissions increases with radial distance, the numbers of submissions were standardized and expressed per 100sqkm (c).

**Fig 3:**
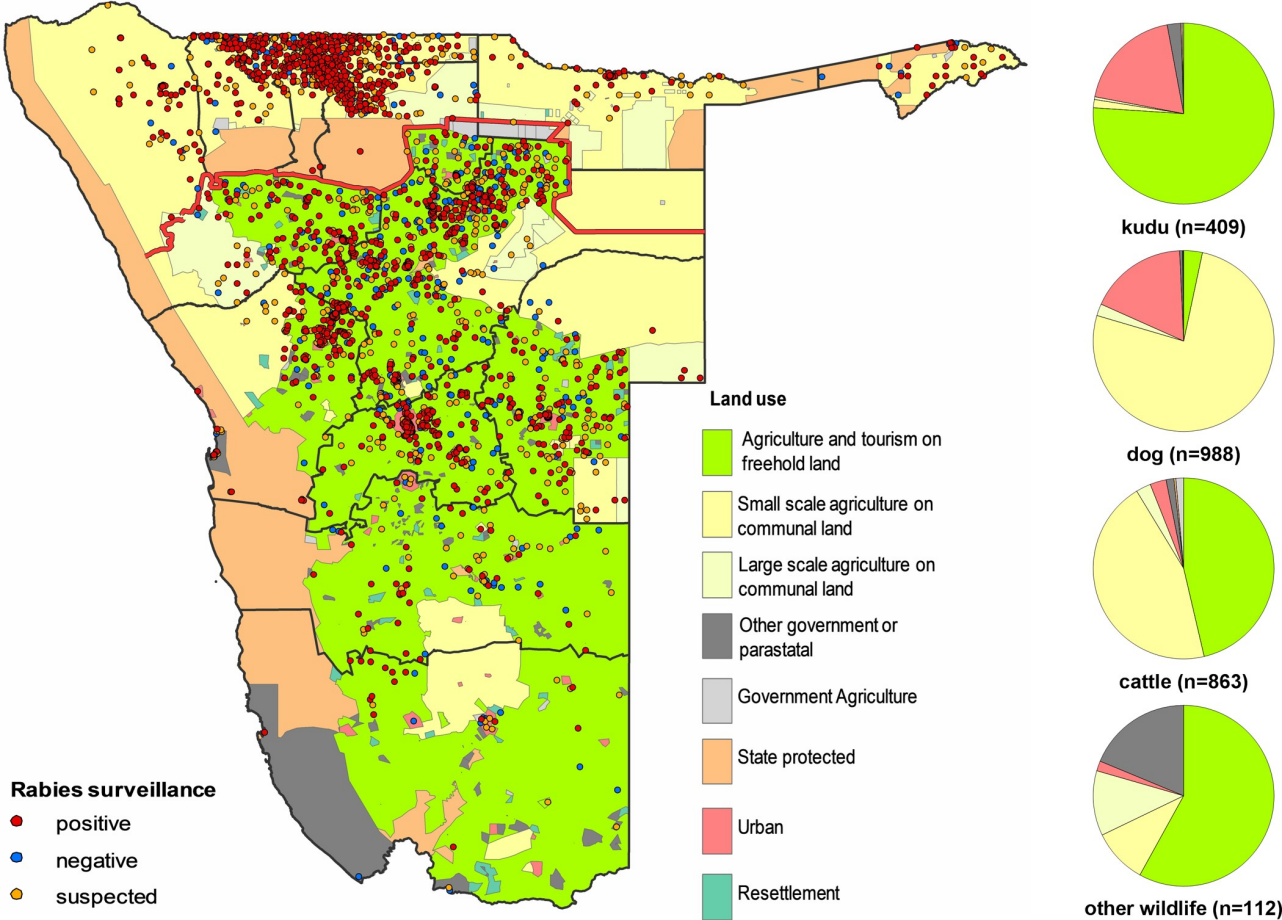
Spatial distribution of rabies surveillance data in Namibia, 2011–2017. Namibian land use is indicated on the map (left), and the proportion of submissions in relation to land use for kudu, dogs, cattle and other wildlife is indicated (right).

After transformation of datasets and correction of missing information, the database for the time period 2011–2017 comprised 4,573 individual datasets. Samples were submitted from all of the 14 Namibian regions (Figs 2A and 3). However, there are certain areas within the country, from where no suspect samples were submitted nor were rabies suspect cases reported to the DVS. In general, the number of submissions were higher from areas closer to one of the two diagnostic laboratories, with this trend being more pronounced for the NCAs (Fig 2B and 2C). However, when normalized against the population per region, the intensity of surveillance was highest in Otjozondjupa (Fig 2A), whereas the lowest values were seen in the northeast (Kavango and Zambesi) and the south (Karas).

The vast majority of dog submissions (77%) originated from communal farm land, followed by urban areas (17%). Only a small fraction (3%) was submitted from freehold farm areas (Fig 3). Kudu and eland submissions were almost exclusively from freehold farmland (76%) and urban areas (19%), whereas the submission of cattle samples is evenly distributed among freehold farms (46%) and communal farm land (46%; Fig 3).

In the frame of this study, a total number of 2,836 animal samples were laboratory-tested on the suspicion of rabies, of which 1,907 (67%; 95% CI: 66%– 69%) tested positive and 929 negative, respectively. The positivity rate of species with submission numbers above 40 was highest for kudu (89%; 95% CI: 85%– 92%) and jackals (*Canis mesomelas*, 87%; 95% CI: 75%– 94%), while cats (54%; 95% CI: 45%– 63%) and dogs (64%; 95% CI: 61%– 67%) demonstrated a relatively lower positivity (Table 1). Generally, the univariate analysis showed no significant difference in the positivity rate beween all samples from the NCAs versus all samples from south of the VCF, whereas subgrouping within animal species revealed higher positivity rates in the northern region than in the south region, except for kudus and elands which only occur in the south of Namibia. While the positivity rate increased from 2011 until 2014, from 2015 onwards a negative trend could be oberserved both in the north and south and across species except elands and jackals. As for the season, significantly lower positivity rates were reported in the dry season than in the rainy season (62% vs. 72%, p<0.001) (Table 1). As per species, significant differences were seen for eland (48% vs. 96%, p<0.001), kudu (81% vs. 94%,p<0.001), cats (42% vs. 69%, p = 0.005), dogs (59% vs. 69%, p<0.001), and cattle (62% vs. 70%, p = 0.01), whereas the trend was not significant for goats. On the opposite, positivity rates in jackals were higher in the dry season, albeit below the level of significance (93% in dry vs. 77% in rainy season, p = 0.170). It should be noted, however, that the sample size for jackals was relatively low compared to other animal species.

**Table 1:** Temporal distribution of rabies surveillance in Namibia for species with total samples above 40. The laboratory results for all samples submitted for testing (N) and the positives thereof (n+) for the years 2011–2017, stratified by geographic region (north, south) and season are shown. For the details of the species encompassing other, please see S2 Table.

Year	2011 N = 341	2012 N = 311	2013 N = 407	2014 N = 304	2015 N = 414	2016 N = 559	2017 N = 500	Total positivity rate N+ *(%; 95% CI)*	Total N (%)
**species total,** N+ *(%)*									
- dog	101 *(90)*	112 *(93)*	101 *(96)*	85 *(93)*	91 *(67)*	97 *(40)*	57 *(29)*	644 *(64; 61–67)*	1007 *(35)*
- cattle	68 *(60)*	82 *(85)*	113 *(82)*	101 *(89)*	107 *(72)*	72 *(44)*	49 *(41)*	592 *(67; 63–70)*	890 *(31)*
- kudu	38 *(97)*	33 *(97)*	92 *(95)*	41 *(100)*	43 *(90)*	28 *(70)*	49 *(75)*	324 *(89; 85–92)*	364 *(13)*
- goat	14 *(61)*	14 *(67)*	22 *(100)*	21 *(100)*	25 *(74)*	18 *(47)*	17 *(44)*	*131 (66; 59–72)*	198 *(7)*
- cat	9 *(90)*	17 *(100)*	8 *(100)*	14 *(100)*	5 *(36)*	5 *(23)*	4 *(13)*	*62 (54; 45–63)*	115 *(4)*
- eland	2 *(50)*	3 *(100)*	6 *(86)*	5 *(100)*	9 *(75)*	5 *(56)*	5 *(71)*	*35 (75; 60–85)*	47 *(2)*
- jackal	11 *(79)*	6 *(86)*	11 *(100)*	3 *(75)*	3 *(100)*	3 *(75)*	4 *(100)*	*41 (87; 75–94)*	47 *(2)*
- other	12 *(48)*	9 *(75)*	15 *(75)*	13 *(87)*	12 *(60)*	9 *(23)*	8 *(22)*	*78 (46; 39–54)*	168 *(6)*
**Total,** N+ *(%)*	**255 *(75)***	**276 *(89)***	**368 *(90)***	**283 *(93)***	**295 *(71)***	**237 *(42)***	**193 *(39)***	**1907 *(67; 66–69)***	**2836**
**region = north**									
species, N+ *(%)*									
- dog	94 *(96)*	105 *(98)*	90 *(100)*	80 *(99)*	86 *(71)*	90 *(40)*	55 *(32)*	600 *(67; 64–70)*	895 *(56)*
- cattle	29 *(85)*	42 *(98)*	48 (94)	37 *(97)*	51 *(70)*	51 *(54)*	35 *(47)*	293 *(72; 67–76)*	408 *(25)*
- goat	8 *(73)*	11 *(79)*	15 *(100)*	16 *(100)*	19 *(70)*	13 *(46)*	12 *(55)*	94 *(71; 62–78)*	133 *(8)*
- cat	7 *(100)*	14 *(100)*	7 *(100)*	12 *(100)*	4 *(36)*	5 *(36)*	3 *(13)*	52 *(58; 48–68)*	89 *(6)*
- jackal	8 *(89)*	2 *(100)*	7 *(100)*	3 *(100)*	1 *(100)*	2 *(100)*	0	23 *(96; 80–99)*	24 *(2)*
- other	6 *(67)*	6 *(100)*	4 *(100)*	5 *(100)*	9 *(75)*	3 *(30)*	4 *(40)*	37 *(66; 53–77)*	56 *(4)*
**North,** N+ *(%)*	**152 *(90)***	**180 *(97)***	**171 *(98)***	**153 *(99)***	**170 *(69)***	**164 *(44)***	**109 *(36)***	**1099 *(68; 66–71)***	**1606**
**region = south**									
species, N+ *(%)*									
- dog	7 *(50)*	7 *(50)*	11 *(73)*	5 *(50)*	5 *(39)*	7 *(32)*	2 *(8)*	44 *(39; 31–49)*	112 *(9)*
- cattle	39 *(49)*	40 *(75)*	65 *(76)*	64 *(85)*	56 *(75)*	21 *(31)*	14 *(30)*	299 *(62; 58–66)*	482 *(39)*
- kudu	38 *(97)*	33 *(97)*	92 *(95)*	41 *(100)*	43 *(90)*	28 *(70)*	49 *(77)*	324 *(89; 86–92)*	363 *(30)*
- goat	6 *(50)*	3 *(43)*	7 *(100)*	5 *(100)*	6 *(86)*	5 *(50)*	5 *(29)*	37 *(57; 45–68)*	65 *(5)*
- cat	2 *(67)*	3 *(100)*	1 *(100)*	2 *(100)*	1 *(33)*	0	1 *(17)*	10 (38; 22–57)	26 *(2)*
- eland	2 *(50)*	3 *(100)*	6 *(86)*	5 *(100)*	9 *(75)*	5 *(56)*	5 *(71)*	35 *(74; 60–85)*	47 *(4)*
- jackal	3 *(60)*	4 *(80)*	4 *(100)*	0	2 *(100)*	1 *(50)*	4 *(100)*	18 *(78; 58–90)*	23 *(2)*
- other	6 (38)	3 *(50)*	11 *(69)*	8 *(80)*	3 *(38)*	6 (21)	4 (15)	41 *(37; 28–46)*	112 *(9)*
**South,** N+ *(%)*	**103 *(60)***	**96 *(77)***	**197 *(85)***	**130 *(87)***	**125 *(74)***	**73 *(39)***	**84 *(43)***	**808 *(66; 63–68)***	**1230**
**Season,** N+ *(%)*									
- January—June	121 *(66)*	158 *(87)*	*220* (91)	143 *(91)*	168 *(94)*	162 *(52)*	108 *(46)*	1080 *(72; 70–75)*	**1494**
- July—December	134 *(84)*	118 *(91)*	148 *(90)*	140 *(96)*	127 *(54)*	75 *(31)*	85 *(32)*	827 *(62; 59–64)*	**1342**

In addition we performed multivariate analyses to control for potential confounders. The logistic regression revealed the significant effect of season (rainy vs. dry), years of investigation (2011 until 2017) and animal species: In the dry season, significantly fewer positive cases were reported than in the rainy season (Table 2). Furthermore, year of laboratory testing and animal species were independently associated with rabies: Since 2015 there was decreasing tendencies in positivity rates in both regions. When considering the animal species separately for each region, it is generally apparent that in the southern regions samples from cattle, kudus, elands, goats and jackals are significantly more likely to test rabies positive compared to samples from dogs. In the NCAs this trend is similar, although not significant. Here, only cats had slightly lower positivity rates than dogs (Table 2).

**Table 2:** Region-stratified logistic regression analysis.

Region	North (N = 1606)				South (N = 1230)			
	% pos	OR	(95% CI)	P	% pos	OR	(95% CI)	P
**Animal species**								
Dog	67	1	-		39	1	-	
Cat	58	0.52	(0.28–0.95)	0.036*	38	1.42	(0.52–3.78)	0.483
Cattle	72	1.35	(0.99–1.84)	0.058	62	1.94	(1.21–3.11)	0.006**
Kudu	-	-	-		89	14.15	(8.19–24.93)	<0.001***
Eland	-	-	-		74	4.85	(2.16–11.46)	<0.001***
Goat	71	1.17	(0.72–1.92)	0.515	57	2.47	(1.25–4.93)	0.009**
Jackal	96	3.21	(0.57–61.03)	0.281	78	7.18	(2.44–24.57)	0.001***
Other	66	0.84	(0.41–1.72)	0.633	37	0.99	(0.54–1.81)	0.974
**Season**								
Rainy	74	1	-		70	1	-	
Cold dry	66	0.56	(0.41–0.76)	<0.001***	55	0.49	(0.34–0.69)	<0.001***
Hot dry	59	0.39	(0.28–0.54)	<0.001***	64	0.70	(0.48–1.02)	0.060
**Year**								
2011	90	1	-		60	1	-	
2012	97	2.91	(1.15–8.35)	0.031*	77	2.34	(1.35–4.11)	0.003***
2013	98	5.49	(1.78–24.03)	0.008**	85	3.22	(1.96–5.36)	<0.001***
2014	99	8.79	(2.43–56.40)	0.004**	87	4.89	(2.73–9.09)	<0.001***
2015	69	0.23	(0.12–0.40)	<0.001***	74	2.08	(1.26–3.46)	0.004**
2016	44	0.07	(0.04–0.12)	<0.001***	39	0.39	(0.25–0.63)	<0.001***
2017	36	0.05	(0.03–0.10)	<0.001***	43	0.38	(0.24–0.61)	<0.001***

Abbreviations: OR, odds ratio; CI, confidence interval; % pos, percent positivity rate from univariate analysis; P, p value; Rainy season: Januar-June, Cold dry: July-September, Hot dry: October-December

Applying a spatial scan statistic to the surveillance data of all animal species, a cluster centered in northwestern Namibia (Relative Risk (RR): 1.32, p = 0.00013) was identified. While the location of the aforementioned cluster changed marginally when dogs were considered exclusively (RR = 1.35, p = 0.0000014), and when they were discarded from the dataset altogether (RR = 1.29, p = 0.012), no significant clusters were found for either kudu or cattle data.

### Rabies in dogs and livestock

**Fig 4:**
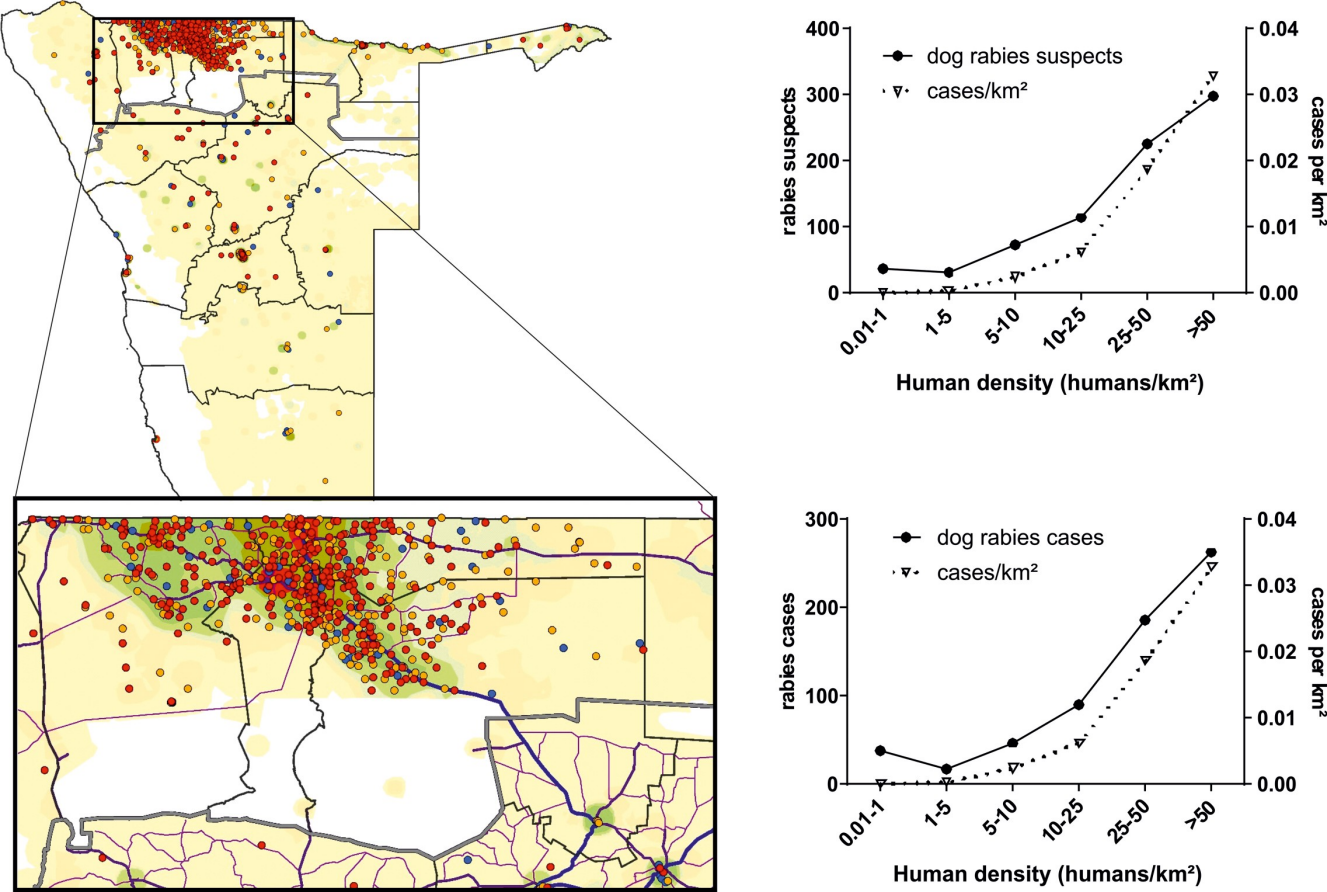
Rabies surveillance in dogs: Rabies cases (red dots), reported rabies suspects (orange dots) and dog samples tested negative (blue dots). The vast majority of rabies cases in dogs were reported from the NCAs north of the veterinary fence (n = 600), while in the remaining part of Namibia only 44 cases were confirmed. The underlying shading represents the relative human population density with darker colors indicating a higher density. Below: Enlargement of the area in the NCAs with the highest numbers of rabies cases. Main roads (blue, pink) are indicated. To the right: charts showing the absolute number of rabies cases or rabies suspects per density class (left y-axis) and the density of rabies cases or rabies suspects per km² per respective density class (right y-axis).

With 644 cases (Table 2), dogs accounted for one third (34%) of all diagnosed rabies cases. The great majority (82%) of dog rabies cases occurred in Omusati, Oshana, Oshikoto, and Ohangwena, but individual cases were also diagnosed in dogs from most other regions of Namibia. There is a strong correlation between human density and the incidence of dog rabies, as both the number of confirmed rabid dogs and reported suspects as well as the relative numbers per square kilometer increased with increasing human population density (Fig 4).

**Fig 5:**
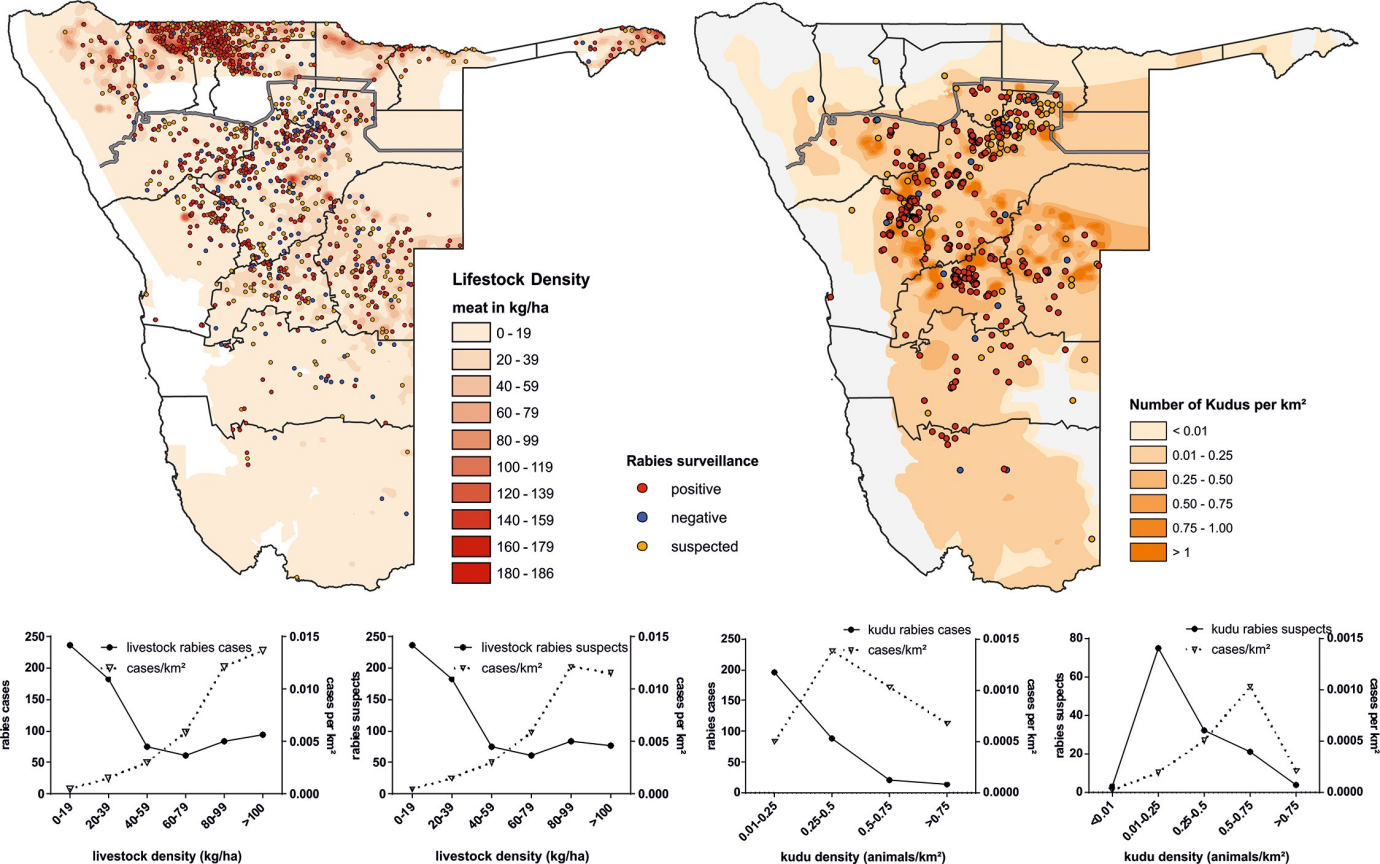
Spatial display of surveillance data (2011–2017) for livestock animals (left), kudus (right). Densities of kudus and livestock are indicated. Below: charts showing the absolute number of rabies cases or rabies suspects per density class (left y-axis) and the density of rabies cases or rabies suspects per square kilometer per respective density class (right y-axis).

In contrast to the clustering of dog rabies in the NCAs, rabies in domestic ruminants (cattle, sheep, and goats) is present throughout the country. About 58% of ruminant laboratory submissions and suspect cases and 57% of confirmed ruminant rabies cases originate from areas south of the VCF (Fig 5). Here, rabies cases are scattered over a vast territory with a concentration of cases in the districts of Khomas, Omaheke, Erongo, Kunene and Otjozondjupa, with only single cases identified in the southern region of Karas.

Generally, and similar to the situation in dogs (here, the human density is a proxy for dog density), rabies cases in livestock animals increase with increasing livestock density (Fig 5). However, this influence is mainly based on the numbers reported in the NCAs, whereas this trend is not obvious when only data from freehold farmland is considered. In contrast to livestock, rabies cases in Kudu are not positively linked with population density (Fig 5).

### Rabies in wildlife animals

Between 2011 and 2017, rabies cases in wildlife were detected in 17 different species (Supplementary S1 Table). Most rabies cases in wildlife were detected in kudu (N = 324), followed by cases in jackals (N = 41) and eland antelopes (*Taurotragus oryx*, N = 35). Sporadically, cases were also detected in other carnivores, i.e. bat-eared foxes (*Otocyon megalotis*; N = 6), aardwolf (*Proteles cristata*; N = 2), African wild cat (*Felis lybica cafra;* N = 2), the African wild dog (*Lycaon pictus*, N = 1) and leopard (*Panthera pardus*; N = 1) and other antelope species, i.e. waterbuck (*Kobus ellipsiprymnus*, N = 3), blue wildebeest (*Connochaetes taurinus*; N = 1), Kirk’s dik-dik (*Madoqua kirkii*; N = 1), oryx antelope (*Oryx gazella;* N = 1), and roan antelope (*Hippotragus equinus*; N = 1, see supplementary S1 Table for the full list of species). The great majority of wildlife rabies cases were reported from freehold farmland south of the VCF where also cattle cases are reported (Fig 5). Rabid jackals were confirmed in many parts of Namibia, although only sporadically from southern regions.

## Discussion

Rabies surveillance should be an integral part of any control programme, e.g. to assess the burden of disease, the effect of the control programme and the association with human medical intervention and post exposure prophylaxis. Given the size of Namibia and the extremely low population density in parts of the country, the analyzed data generally indicate a fairly good level of surveillance, albeit with variations when considering the human population (Fig 2). In fact, with an average of 26 submitted samples per 100,000 people per annum, Namibia’s surveillance is comparable to the US (31; [34]), and much more intensive than for example Turkey (1.4; [35]). Unfortunately, data concerning the number of tested animals is scarce for Africa and therefore it is not possible to compare Namibia’s surveillance to other African countries.

Obviously, there are areas within the country, where rabies-suspect animals may not have been detected, and hence not reported to the DVS. Thus, no region in the country would fulfill criteria to be considered free of the disease. Another constraint for adequate surveillance is the logistical access to laboratories. Although the CVL in Windhoek has a central geographical position (Fig 1), it may still be difficult to transport specimens, i.e. heads of suspected animals, for more than half a day appropriately under tropical climatic conditions. In the NCAs, the laboratory in Ondangwa receives samples mainly from the surrounding areas which also have the highest human population densities and thus dog populations, but generally, submissions were more frequent from areas closer to one of the two diagnostic laboratories (Fig 2B and 2C), which may also be affected by infrastructure, particularly road networks (Fig 4). It is thus likely that rabies in rather sparsely populated areas with limited access to infrastructure is under reported and samples are not submitted. As a consequence, the most important aspect and a cornerstone of the Namibian Rabies Control Strategy is education on all levels [24]. On the other hand, surveillance intensity also seems to be influenced by disease incidence, e.g. there are more samples submitted from sparsely populated areas in Kunene than in Karas (Fig 2A).

The analysis of human rabies data confirms the burden of the disease in the NCAs. Highest numbers were reported from the Kavango region, from the hospital in the city of Rundu. Interestingly, this does not correlate to the clustering of dog rabies cases in the central areas of the NCAs. Presumably, the reasons for the human cases are lack of awareness, education and the difficulties in reaching medical intervention for PEP. The same circumstances may result in a less-than efficient animal rabies surveillance (Fig 2A). In fact, Kavango and the neighboring Zambezi region are amongst the poorest and less developed regions of Namibia [36]. The human rabies annual incidence per 100,000 people in the NCAs ranges between 0.8 and 2.6 and appears similar to values estimated for other countries in southern Africa [3, 37]. Because all human cases are based on clinical observations, the true incidence is likely to be even higher as some rabies patients die at home instead of in a healthcare facility where the cases are recorded, and rabies may be missed as other diseases like malaria may cause similar symptoms in humans [38]. In recent years, cases started declining from 2016 onwards, possibly due to the rabies project that was implemented in the northern communal areas in early 2016. Also, the number of animals which tested negative increased from 2015 onwards. In the future it is envisaged to improve the accuracy of human rabies surveillance by laboratory confirmation *ante* and *post mortem* where possible. Additionally, verbal autopsy, a research method that helps determine probable causes of death in cases where there was no medical record or formal medical attention [39], could be used to further assess causes of death in communities with limited access to medical infrastructure, as was demonstrated for neurological diseases including rabies in other countries [40].

As for wildlife surveillance in Namibia, also a rather heterogeneous level is obvious. The vast majority of samples originated from freehold farm areas. Here, farmers have the possibility to observe suspect animals, and have the interest and resources to submit samples for laboratory confirmation. Interestingly, in the Kunene region where both cattle and kudus occur, only cattle are submitted for testing (Figs 3 and 4). Obviously, for the pastoral farmers control of diseases in their livestock is crucial to sustain livelihood, while wildlife does not play an important role and hence these samples are likely not to be submitted. Generally, in nature conservancies, detection of diseased animals is less likely which results in only sporadic submissions (Fig 3). Another aspect is the fact that due to their limited visibility and their rapid disappearance after death by scavengers, surveillance in small carnivorous species like jackals is particularly difficult.

The observed distinction between dog-mediated rabies in the NCAs versus wildlife mediated rabies in central and southern regions remains stable as it had been described earlier [9, 18]. It is evident from the analyses that the domestic dog is the major reservoir and vector of the disease in the NCAs. Here, a clear correlation with the human population density as a proxy for dog density was observed (Fig 4).

The situation in central and southern Namibia is less clear. Under the prevailing assumption that there is horizontal transmission between kudus [17], the fact that besides the kudu, also cattle and elands, among others, die of rabies in spatial and temporal vicinity is difficult to explain (Figs 3 and 4). Although the latter two species are closely related to the kudu [13], experimental horizontal transmission of kudu rabies virus to cattle was not successful [19]. Also, even though transmission among bovids was possible, this would not explain cases in other species. Rather, wild canid species, e.g. jackals and bat eared foxes, are more likely to act as reservoirs and infect both wildlife ruminants and livestock in the same area. In fact, in earlier studies it was reported that rabid jackals were often encountered at watering sites where they attacked cattle and near farm buildings where they also attacked other domestic animals and humans [41]. Eventually, these infections may lead to an onward transmission from kudu to kudu [23].

The circulation of rabies virus within wildlife canids may explain the fairly high number of dogs from this area that died of rabies (Fig 4). Vaccination of dogs is compulsory, but the cases indicate limited compliance. An alternative explanation, i.e. that dog rabies south of the VCF is linked to the endemic areas in the NCAs, is highly unlikely as the distance is too long for natural movement, and translocation by humans is not common. As rabid jackals have been detected in all parts of the country, it seems evident that jackal or other carnivore-mediated wildlife rabies is endemic all across the country. The observed seasonality of rabies cases, i.e. the significantly higher positivity rate in the first half of the year, the rainy season, is striking. This trend was observed for dogs, livestock animals and wildlife. As for the latter, historically, it was shown that kudu cases peaked in March and July [18], which is partly in agreement with our observations. With incubation periods ranging from 12–250 days, diseased animals could have been infected already in the hot dry season (September–December) or in the rainy season (January-June). Interestingly, in Zimbabwe, under very similar ecological circumstances, the incidence of reported jackal rabies peaked during June to August and December to May, and was explained by the social behavior and population dynamics of jackals [42, 43]. For dogs and the association to livestock in the NCAs, the trend may be a result of the pastoral seasonal movement of livestock including guarding dogs. Also, dogs may be temporarily abandoned thus increasing the free roaming and potential interaction with infectious animals.

Rabies pathogenesis is distinctive, as the animals will show more or less specific clinical signs and will eventually die of the disease. Therefore, surveillance activities should focus on indicator animals that need to be confirmed by laboratory tests [44]. The overall positivity rate of submitted samples being >65% in Namibia is similar to observations in South Africa [45] and Zimbabwe [43, 46]. In other parts of the world with endemic rabies, this figure is much lower. While around one quarter of submissions tested positive under a passive surveillance system in Brazil [47], only between 12–24% of wildlife samples were rabies positive in the USA [34]. This may in part be due to the fact, that the latter surveillance systems are more advanced insofar that more samples are available for testing, not just indicator or suspect animals like in Namibia. However, in domestic animals this should not differ significantly between countries. Therefore, the positivity rate of 66% in cattle (95%CI: 63–70) is almost twice as high as the figure (38.6%, 95%CI: 35.9–41.4) from an epidemiological study on cattle rabies in Turkey [48]. The highest positivity rate of 87% (95%CI: 75–94) was seen in jackals. It seems that other etiologies for encephalitis, e.g. canine distemper may not be so prevalent in southern Africa. In fact, rabies was a major cause of death in Zimbabwe [43] and in the South African Limpopo province where also two thirds of submitted jackals tested positive for rabies [49]. A very high rate was also found for kudus (Table 2). It is known that clinical signs, particularly for kudu rabies, seem to be pathognomonic [18], and therefore it is likely that only those are submitted for testing. On the other hand, if the disease is confirmed once, there may be a certain reluctance for further submission of samples, thus leading to underreporting of the disease in kudu.

While, generally speaking, rabies surveillance appears adequate to demonstrate the presence of the disease in many areas, there is still room for improvements. The number of submissions could be increased by providing kits for easier sampling, storage and transport [50]. Particularly concerning animals from freehold farms, samples could be safely stored in a freezer and eventually be transported to the CVL, accompanied by the official DVS sample submission form. Notification of rabies currently only covers rabies suspects in dogs, cats, wild carnivores, cattle, sheep, goats, pigs, horses and donkeys [Government Notice 180 of 2013], but not in wild herbivores, which may therefore be underreported and should be included in the list. As in many other countries, surveillance and laboratory testing of suspects in Namibia only confirms or rules out rabies. However, about one third of all submitted animals tested negative for rabies but had shown clinical signs or even had died of an unidentified disease. If sufficient resources are available, the samples tested negative for rabies could be screened with a set of multiplex–PCRs targeting either syndromes [51] or animal species specific diseases [52]. Eventually, such samples could also be subjected to untargeted genome analyses using next generation sequencing [53]. In fact, a recent study from rabies-free Switzerland demonstrated the prevalence of neuro-infectious diseases in fallen cattle, particularly in those with neurological disorders [54]. As for dog rabies surveillance, education should result in higher submission rates. With the national rabies control strategy that started in 2015, initially, the number of submissions and thus the number of detected cases are likely to increase, but on the long term, mass dog vaccination should result in a significant reduction of cases as seen in other countries, e.g. in Southern Africa [55, 56] and particularly in Latin America [57].

Our study had several limitations: inevitably by nature, surveillance for rabies is not a random sampling but targeting clinically affected or dead animals [58], thus representing a challenge for statistical analyses [59]. As pointed out above, there are other factors like disease awareness, logistical hurdles, etc. that may influence the likelihood of a sample being submitted for laboratory investigation [1]. Nonetheless, for the first time in this study, we have linked rabies surveillance data with available data e.g. on population densities of humans and animal species to uncover some complex interactions among spatial data. Of note, some of these secondary data files originate more than a decade ago (S2 Table). Unfortunately other more current data was not available and but is quite conceivable, that the fundamental dimensions of these data have not changed much over the years. Also, the open access and availability of these GIS data is exceptional, particularly for Africa.

### Conclusions

The analysis of both human and animal rabies surveillance data from Namibia in combination with other available data show that human rabies cases in the northern regions are linked to poor infrastructure, the latter also being likely to contribute to inadequate surveillance (Fig 2A).

In comparison to previous reports on rabies surveillance in Namibia [18], decades of efforts were so far insufficient to control rabies in dogs. Namibia now takes responsibility for its people and strives to reduce dog-mediated human rabies by education, proper and timely administration of PEP and, most importantly, by controlling the disease in its main reservoir, the dog through repeated mass rabies vaccination aiming at 70 percent coverage [24], in concordance with the recently adopted global strategic plan [60]. To this end, surveillance and subsequent epidemiological analyses will be essential to demonstrate the effect of this control programme.

## Supporting information

S1 Table Sources for the Namibian GIS layers. (XLSX)

S2 Table Animal rabies surveillance data for the years 2011–2017. (XLSX)
